# Modification of Alginates to Modulate Their Physic-Chemical Properties and Obtain Biomaterials with Different Functional Properties

**DOI:** 10.3390/molecules26237264

**Published:** 2021-11-30

**Authors:** Piotr Rosiak, Ilona Latanska, Paulina Paul, Witold Sujka, Beata Kolesinska

**Affiliations:** 1 Institute of Organic Chemistry, Faculty of Chemistry, Lodz University of Technology, Zeromskiego 116, 90-924 Lodz, Poland; piotr.rosiak@dokt.p.lodz.pl (P.R.); paulina.paul@dokt.p.lodz.pl (P.P.); 2 Tricomed S.A., Swietojanska 5/9, 93-493 Lodz, Poland; ilona.latanska@tzmo-global.com (I.L.); witold.sujka@tzmo-global.com (W.S.)

**Keywords:** carboxylic group transformations, reactions of hydroxyl functions, degree of substitution (DS), regio- and chemoselectivity and specificity, hydrogelation, alginate peptide conjugates, diversified biological activity of alginates

## Abstract

Modified alginates have a wide range of applications, including in the manufacture of dressings and scaffolds used for regenerative medicine, in systems for selective drug delivery, and as hydrogel materials. This literature review discusses the methods used to modify alginates and obtain materials with new or improved functional properties. It discusses the diverse biological and functional activity of alginates. It presents methods of modification that utilize both natural and synthetic peptides, and describes their influence on the biological properties of the alginates. The success of functionalization depends on the reaction conditions being sufficient to guarantee the desired transformations and provide modified alginates with new desirable properties, but mild enough to prevent degradation of the alginates. This review is a literature description of efficient methods of alginate functionalization using biologically active ligands. Particular attention was paid to methods of alginate functionalization with peptides, because the combination of the properties of alginates and peptides leads to the obtaining of conjugates with properties resulting from both components as well as a completely new, different functionality.

## 1. Alginates General Information and Their Structure 

Alginate was discovered in 1881 by Stanford [1]. The term is usually used to refer to salts of alginic acid, although it can refer to alginic acid and all its derivatives [2]. Alginates are unbranched polysaccharides linked by 1→4 glycosidic bonds composed of β-d-mannuronic acid (M) and its C-5 epimer α-L-guluronic acid (G) [3]. These polysaccharides are an important building block in algae and exopolysaccharide bacteria, including *Pseudomonas aeruginosa*. Only alginates isolated from algae are commercially available. The natural origin of alginates causes variation in their biopolymer compositions, sequences, and molecular weights, depending on the source and species used by the manufacturer. Industrial production of alginates amounts to about 30,000 tons per year, which is less than 10% of the available bio-synthesized polymer [4]. Modifying the properties and expanding the spectrum of possible applications could lead to wider use of this relatively widely available raw material.

Combining the methods and tools of chemistry and biochemistry gives access to modified alginic acid derivatives with controlled properties resulting from the location, nature, and quantity of the introduced substituents. This allows for modulation of their solubility, hydrophobicity, affinity for specific proteins, and many other properties. The modification process is, in many cases, complicated by the variable properties of the starting material (alginic acid), such as its solubility, pH sensitivity, and structural complexity. Therefore, it is necessary to control the course of the chemical reactions and to analyze the final compounds. Despite the difficulties involved, there is much interest in the modification of alginates leading to their controlled derivatization.

The importance of alginates as natural polysaccharides in medicine cannot be overstated. One of their most important applications is the use of cross-linked alginates to produce hydrogels for cell encapsulation [5,6,7]. A flagship example is the use of alginate gels to encapsulate islets of Langerhans for diabetes treatment [8]. Alginates are widely used as dressing materials to treat various types of wounds [9]. However, they intensify the effects of cystic fibrosis, because alginate gels secreted by *Pseudomonas aeruginosa* form bacterial biofilms [10].

This literature review discusses methods used to modify alginates. Alginate modifications may have one of two objectives: (1) to introduce completely new properties not found in unmodified alginates or (2) to improve their existing properties.

### The Structure of Alginates

**Figure 1:**
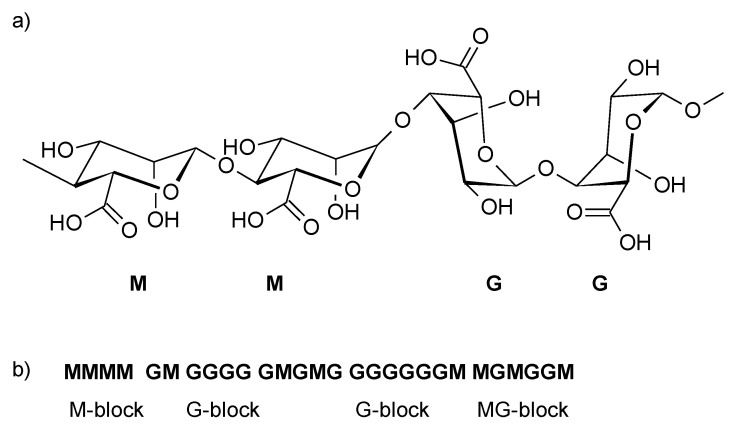
Structure of alginates: (**a**) conformation of the oligosaccharide chain; (**b**) distribution of M and G blocks in the polysaccharide chain.

The structure of alginates was established based on partial hydrolysis and subsequent fractionation [11]. Fractionation leads to a soluble (hydrolysable) fraction and an insoluble fraction (resistant to hydrolysis). The insoluble fractions consist of molecules composed mainly of d-mannuronic acid (M) residues or L-guluronic acid (G) residues. The soluble fractions are rich in compounds containing alternating MG residues [12]. There are considerable differences between alginates in terms of the sequence of G, M, GG, MM, and GM/MG blocks (Figure 1) and of their chemical compositions, which may depend on the species of algae and even the time of year they were harvested. The calculated dry weight content of alginates is in the range of 17–44% [13,14]. The structure of M, G, and MG blocks is determined based on an examination of ^1^H-NMR and ^13^C-NMR [15,16]. 

The results of fractionation of alginate hydrolysis products have been confirmed by computer modeling, providing better understand the alginate microstructure [17,18,19].

## 2. Modulating the Physical Properties of Alginates

The physical properties of alginates depend on the relative proportions in the biopolymer of the M and G blocks. The solubility of alginates in water depends on the pH of the aqua solution, the presence of a cosolvent, the ionic strength of the dissolving medium, the presence of gelling-promoting ions, and the structural factors of the biopolymer. To dissolve alginates requires a pH above the critical value at which deprotonation of the carboxyl groups occurs. Changing the ionic strength of the medium affects the properties of the solution, such as polymer conformation, chain elongation, viscosity, and solubility.

Alginates can be made soluble in organic solvents by the formation of tetrabutylammonium (TBA) salts on the carboxyl groups of the polysaccharide. It is possible to completely dissolve TBA-alginate in polar aprotic solvents containing tetrabutylammonium fluoride (TBAF) [20]. The solubility of alginates depends on the structure and hence the properties of the carboxyl groups in the polymer backbone. When its carboxyl groups are in protonated form, alginic acid is not fully soluble in any solvent system, including water. Sodium alginate is soluble in water, but not in any organic solvent. TBA alginate salts are soluble in water, ethylene glycol, and polar aprotic solvents containing TBAF [21] (Table 1).

**Figure 2:**
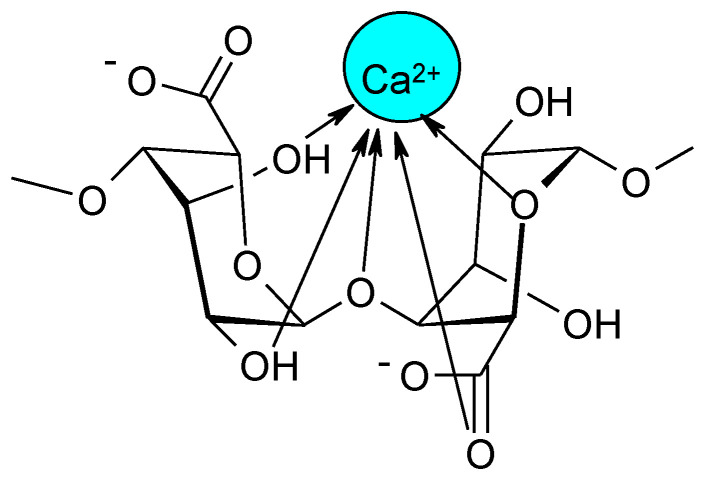
Probable structure of the complex formed by binding of the Ca^2+^ ion with L-guluronic acid residues.

**Figure 3:**
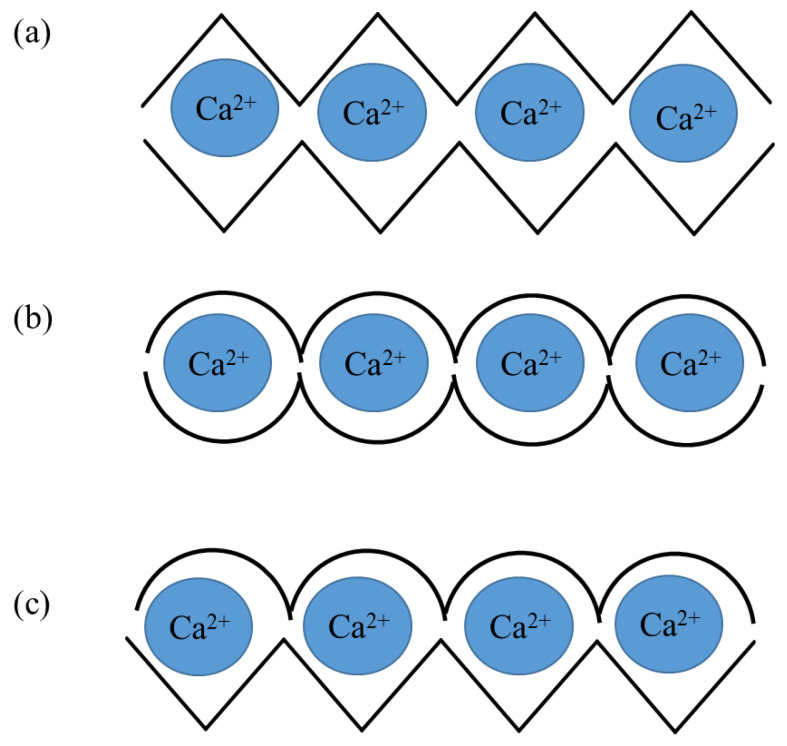
P Possible structures of alginate chelates with Ca^2+^: (**a**) GG/GG, (**b**) MG/MG, (**c**) GG/MG.

A characteristic feature of alginates is their ability to chelate divalent cations, leading to the formation of hydrogels. Gel formation is caused by interactions between G blocks, which bind to form tightly interacting nodes (ionic cross-linking, cross-linking) in the presence of divalent cations [22]. Apart from G blocks, MG blocks also take part in ion cross-linking, leading to relatively weaker supramolecular structures [23]. Thus, alginates with a higher G content form more stable hydrogels. The affinity of alginates to divalent ions is as follows: Pb > Cu > Cd > Ba > Sr > Ca > Co, Ni, Zn > Mn [24]. Ca^2+^ is the most commonly used cation for the formation of alginate gels. Depending on the number and length of M and G blocks, the mechanical properties of the alginate and its ability to form gels change [25]. By reacting the carboxyl group of α-L-guluronic acid with a divalent metal ion, it is possible to enclose the ion in the space between two adjacent meric units, which ultimately leads to the formation of an “egg-box” structure (“egg in an egg box”) (Figure 2 and Figure 3). This structure makes it difficult to exchange calcium ions with sodium ions. Therefore, if a gel is dominated by G blocks it is called a hard gel, whereas gels with a predominance of M blocks are called soft gels [26,27].

Alginates can be cross-linked with calcium ions in two ways. In the diffusion method, cross-linking ions diffuse into the alginate solution from an external reservoir. In the internal setting method, the Ca^2+^ ion source is in the alginate solution and a controlled trigger (typically pH or Ca^2+^ solubility) releases cross-linking ions into the solution. The diffusion method leads to hydrogels with a gradient of Ca^2+^ ion concentrations. The internal setting method leads to hydrogels with a homogeneous concentration of Ca^2+^ ions [28]. In the diffusion method, a sodium alginate solution is usually dropped into the CaCl_2_ solution. In the internal setting method, CaCO_3_ is used as the insoluble Ca^2+^ ion source. The pH change caused by the slowly hydrolyzing d-glucono-δ-lactone (GDL) triggers the release of Ca^2+^ ions, leading to gel formation.

When the pH of the alginate solution is lowered under controlled conditions below the pKa of uronic acid, an acid hydrogel is formed. Acid gels are stabilized by the intermolecular network of hydrogen bonds. There are two known methods of forming of acid gels [29]. In the first method, slowly hydrolyzing GDL lactone is added to the sodium alginate solution. In the second method, the preformed calcium alginate gel is treated with acid, resulting in an exchange of Ca^2+^ ions to H^+^.

An arrangement and composition of β-D-mannuronic and α-L-guluronic acid in alginates influenced on the chemical, physical, and biological properties of the biopolymer. The most useful analytical method that allows obtaining information on the distribution of M, G, and MG blocks and their lengths are techniques based on NMR [30]. In addition, mass spectrometry (MS), based on either electrospray ionization (ESI) or matrix-assisted laser desorption/ionization (MALDI), is a useful technique for carbohydrate analysis to obtain detailed information about the structure, including molecular mass, sugar constituent, sequence, inter-residue linkage position, and substitution pattern [31]. The most commonly used techniques for the study of biopolymers are: Fourier transform infrared spectroscopy (FTIR), X-ray diffraction (XRD), thermogravimetric analysis (TGA), differential scanning calorimetry (DSC), and scanning electron microscopy (SEM) [32,33,34]. 

## 3. Chemical Properties of Alginates and Modification Methods

**Figure 4:**
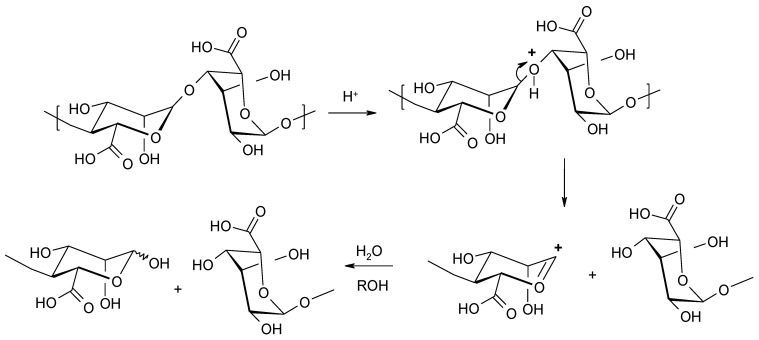
Acid-catalyzed degradation of alginic acid.

Polysaccharides, including alginates, undergo hydrolytic cleavage in an acidic environment [35]. The reaction is performed in three steps: (1) protonating the oxygen atom at a glycosidic bond to form the conjugated acid; (2) hydrolysis of the conjugate to form the non-reducing terminus and the carboxonium ion; and (3) rapid addition of water to the carboxonium ion, leading to the formation of a reducing end (Figure 4). In the form of dry powder, sodium alginate can be stored for several months at room temperature without degradation. The half-life can be extended to years by storage at low temperature. Alginic acid is degraded significantly faster than sodium alginate. This is due to internal acidic catalysis with the participation of the C-5 carboxyl group [36].

**Figure 5:**
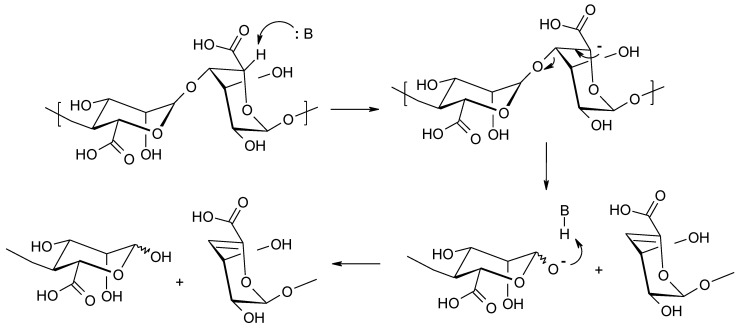
Base catalyzed degradation of alginate (β-elimination).

The enzymatic degradation of alginates in the presence of lyases occurs according to the β-elimination mechanism (Figure 5), ultimately leading to the formation of unsaturated compounds [37,38]. The alginate degradation reaction has a similar course in an alkaline environment. The reaction rate increases rapidly above pH 10.0 and below pH 5.0. In the case of decomposition in an environment with a pH above 10.0, the reaction takes place mainly by β-elimination, whereas in the case of an acidic reaction (pH below 5.0) acid-catalyzed degradation occurs as described above [39]. The β-elimination reaction consists of the cleavage of the proton at the C-5 position, which is supported by the presence of an electron-acceptor substituent (carbonyl group) at the C-6 position [40].

Alginates are prone to degradation not only in the presence of acids or bases, but also in the presence of reducing compounds at neutral pH. Many reducing compounds, such as hydroquinone, sodium sulfite, sodium bisulfide, cysteine, ascorbic acid, hydrazine sulphate, and leuco-methylene blue, also cause alginate degradation [41]. Brown algae alginates contain different amounts of phenolic compounds, depending on the species [42]. The rate of degradation increases with the amounts of phenolic compounds in the alginates. Sterilization techniques, such as high temperature treatment, ethylene oxide treatment, or γ-radiation, cause degradation of alginates [43]. The susceptibility of alginates to degrade must be taken into account when planning chemical modifications of this polysaccharide.

Modifications of alginates may be aimed at improving their existing properties or introducing completely new properties [44]. Derivatization and strategies for alginate modification depend on three main factors: solubility, reactivity, and methods of characterizing new materials. As already mentioned, alginates can be dissolved in water, organic solvents, or mixed systems. The choice of solvent system may dictate the types of reagents that can be used for the modification. Moreover, the degree of solubility of the alginate in the solvent system may affect the selectivity of the modification. Alginates can be modified on two secondary -OH groups (C-2 and C-3) or on the carboxyl group (C-6). The difference in reactivity between the two types of functional groups can be used to selectively modify one of the two types of functional groups. Regioselective modification of the hydroxyl groups at the C-2 or C-3 position is difficult, due to the slight differences in their reactivity. The selectivity of the modification can be additionally controlled by taking advantage of differences in the reactivity of the M or G residues. This can be achieved, for example, by selectively chelating the G residues in Ca-alginate gels or by taking advantage of the partial dissolution properties of alginates in different solvent systems. To more thoroughly understand and predict the effects of modifying new alginate derivatives, it is often necessary to have multiple alginate samples with a varied and fixed M/G ratio. Typically, it is also necessary to derivatize alginates enriched in M, G, or MG blocks to obtain the data necessary to understand the course of the reactions. The lack of commercial availability of sequence-controlled alginates impedes the complete structural characterization of their derivatives. Due to the complex nature of the alginate backbone, advanced analytical techniques are often needed.

### 3.1. Reaction of Hydroxyl Groups

#### 3.1.1. Acylation of Hydroxyl Groups

Chamberlain et al. [45] were the first to describe the acetylation of alginic acid hydroxyl groups. It was observed that the hydroxyl groups in dry alginic acid yarn do not react with acetic anhydride, due to the strong hydrogen bonds between the functional groups. However, upon swelling with water the hydroxyl groups become available for the acetylating agent. After the swelling process, the solvent was exchanged by replacing the water with glacial acetic acid and then reacted with acetic anhydride in the presence of H_2_SO_4_ as a catalyst. The alginate di-acetate was obtained with 97.3% yield (based on total acetyl content). However, the harsh reaction conditions caused significant degradation of the polysaccharide.

Wassermann [46] proposed the use of ketene as an alginate acetylating reagent. This approach eliminated the need to use strong acids or pyridine as esterification catalysts. Acetylation was performed using swollen alginic acid in acetone. The obtained alginic acid acetate was insoluble both in water and in typical organic solvents. Alginate sodium and calcium salts have also been acetylated successfully under the conditions described above. Based on the titration results, it was discovered that one acetyl group was introduced onto one monosaccharide residue. The degree of degradation of the polysaccharide chain was determined based on viscometric methods. The acetylated sodium alginate and alginic acid were found to be characterized by reduced viscosity, indicating partial degradation of the polymer.

Schweiger [47] described a method for the preparation of partially or fully acetylated derivatives of alginic acid using classical esterification conditions and an acid catalyst. The reaction was carried out using an alginic acid suspension and a mixture of acetic acid, acetic anhydride, and perchloric acid. The degree of substitution (DS) was as high as 1.85 when the reaction was run at moderately high temperature. This method did not cause significant degradation of the polysaccharide. In alginate acetates with different degrees of substitution, it was possible to study the chelation mechanism based on the structures of the ionically cross-linked alginate gels [47]. It was found that the addition of divalent ions to the alginate diacetate solution (DS = 2.0) does not lead to gel formation. The alginate acetates with DS = 1.4, which contained monoacetylated and diacetylated derivatives (no non-acetylated saccharides), showed a very low tendency to gel. This suggested that the presence of non-acetylated saccharide units is necessary for the formation of chelates and the presence of hydroxyl groups is needed for ionic cross-linking, while carboxylate anions are only marginally responsible for gelation. Based on the collected data, a chelate structure was proposed in which a single Ca^2+^ ion is coordinated with two carboxyl groups and two vicinal hydroxyl groups of the same saccharide.

**Figure 6:**
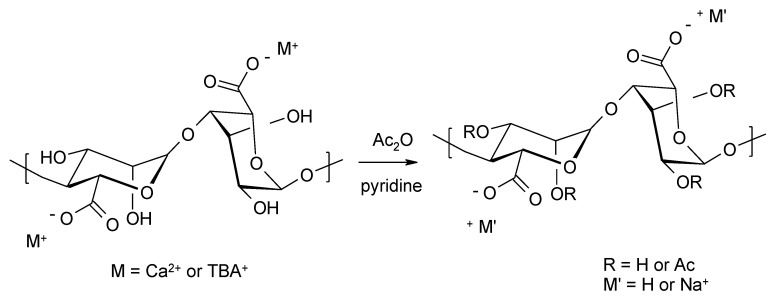
Acetylation of alginate using a mixture of pyridine/acetic anhydride. Gel acetylation: M = Ca^2+^ or TBA^+^. Acetylation of a homogeneous system in DMSO/TBAF, M = TBA^+^, M’= -H^+^ or Na^+^.

A different approach to the acetylation of alginates was proposed by Skjåk-Bræk [48], along with a detailed analysis of the degree of substitution and the structure of final products. The starting materials were calcium alginate gel and soluble tetrabutylammonium salts of alginic acid (Figure 6), which were converted into pyridinium alginate salts before the acetylation process.

Regardless of the reactants used, the presence of water was found to be crucial for the success of the reaction and the DS depended on the amount of water. The maximum DS value was achieved with a water content of 20%. The structure of the obtained alginate acetates was characterized using the ^1^H-NMR technique. Due to overlapping of the anomeric proton signals in the high field, O-acetyl residues with signals at about 2.0–2.2 ppm were used in the structural analysis of the products. To accurately assign the structures to the signals, it was necessary to perform follow-up studies using alginate acetates enriched in G and M blocks. Based on comparative studies, it was found that signals in the range of 2.04–2.06 ppm are derived from the monoacetylated derivative. Using alginates from *Laminaria hyperborean* (*L. hyperborea*) with FG = 0.68 and *Ascophyllum nodosum* with FG = 0.40, the relative susceptibility to esterification of the M and G residues was determined (Table 2).

The results presented in Table 2 clearly show that in the case of acetylation of calcium alginate gels with the pyridine/acetic anhydride mixture, the M residues are more susceptible to acetylation.

The use of the same acetylation method under homogeneous reaction conditions with tetrabutylammonium alginate in selected organic solvents (DMSO, DMF, DMAc, DMI with the addition of TBAF) led to partial acetylation with DS in the range of 0.74–0.85. A DS value of 1.0 was obtained under anhydrous conditions, where TBAF was obtained in situ [49] or by adding additional portions of acetic anhydride. Despite numerous attempts, it was not possible to significantly increase the DS value over 1.0. This can be explained by the influence of electronic effects in the ring of the monosaccharide unit. Only one electron-withdrawing substituent is present in the ring in the starting TBA-uronic acid salt. During acylation, a monoacetyl derivative is formed, which means that the molecule already has two electron-accepting substituents. It has been suggested that the presence of these two substituents significantly reduces the reactivity of the second hydroxyl group. The DS value is therefore limited to ~1.0. Another possible explanation is stereochemistry of the M and G residues. In the case of the M block, the hydroxyl group at position 2 is in the axial position and can interact with the C4 proton through 1,3-diaxial interaction, making acetylation of the hydroxyl group preferred in position 3. In the case of block G, the OH group in position 3 is axially arranged and can interact with a proton at carbon C5 (1,3-diaxial interaction). This gives preference to acetylation of the hydroxyl group in position 2. For both blocks monoacetylation is preferred, resulting in a final DS of 1.0.

The dependences of the DS value on the structure of alginates (with different M/G ratios) and on the acylation reaction conditions have also been studied. It was found that under the conditions of a homogeneous reaction (DMSO-TBAF solvent), regardless of the structure of the substrate, the DS value was almost constant at ~0.8 (Table 3). This indicates that the reaction is non-selective and that the M and G blocks are acetylated.

The differences in DS observed under heterogeneous reaction conditions may result from the different solubility of the polysaccharide, depending on its composition. However, homogeneous reaction conditions were found to favor the degradation of the polysaccharide chain, which was not observed for products obtained under heterogeneous reaction conditions. Polysaccharide degradation in the DMSO-TBAF reaction may be related to the basic nature of the fluoride anion [49].

In many cases the abundant hydroxyl and carboxyl groups on the backbone of alginate (high hydrophilicity, the nature of polyelectrolyte) is an unfavorable feature, that is why the chemical modification of alginate by hydrophobic groups can be considered as an effective way to overcome this drawback. The two secondary C-2 and C-3 hydroxyl groups and the C-6 carboxylic acid groups can be used to obtain alginate esters. Amphiphilic polymers are of increasing interest because macromolecules containing hydrophilic and hydrophobic groups are capable of self-aggregation into micelle-like aggregates with a hydrophobic inner core and a hydrophilic outer shell in an aqueous solution that can be used in medicine as a drug delivery system [50,51]. Additionally, the attaching of hydrophobic segments onto the backbone of alginate can improve properties such as molecular flexibility, hydrophobicity, and physicochemical and biological characteristics, which make it capable of achieving the loading of hydrophobic drugs through its self-aggregation and prolonging its stability in biological medium [52,53].

#### 3.1.2. Phosphorylation of Alginates

**Figure 7:**
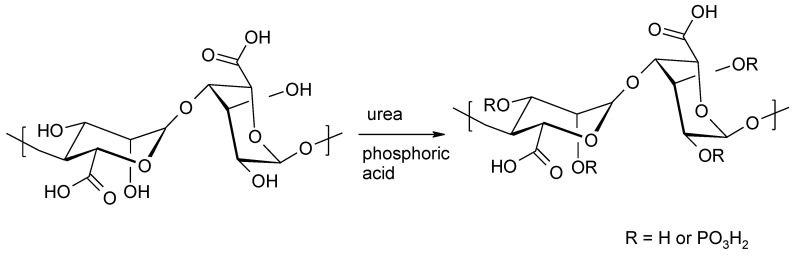
Phosphorylation of alginates.

Phosphorylated alginates are used to induce nucleation and the growth of hydroxyapatite (HAP) [54]. Phosphorylation is carried out with a mixture of urea and phosphoric acid (Figure 7). The maximum DS that has been achieved is 0.26, in a reaction in which the molar ratio of alginate:H_3_PO_4_:urea was 1:20:70. Phosphoric acid is responsible for the observed degradation of the alginates (a 2–4-fold reduction in the molecular weight of the phosphorylated alginate compared to the original polysaccharide).

Based on NMR analysis using ^1^H and ^31^P, ^1^H-^1^H COSY, ^1^H-^31^P HMBC, ^1^H-^31^P HMQC-TOCSY, and ^1^H-^13^C HSQC, it was found that only monomer G is phosphorylated. However, it was not possible to determine whether the reaction takes place on the 3-OH or the 2-OH G units, because the observed correlations in the ^1^H-^31^P-HMQC-TOCSY spectra were too low.

The phosphorylated alginate derivatives were used as a starting material for preparing injectable hydrogels. Results of biological tests showed that the injectable hydrogels demonstrated comparable properties to the pure alginate hydrogel in terms of cytotoxicity and 3D encapsulation of cells. Additionally, it has been found that they have the appropriate physicochemical and mechanical properties for in vivo [55].

#### 3.1.3. Incorporation of Sulphate Residues into Alginates

**Figure 8:**
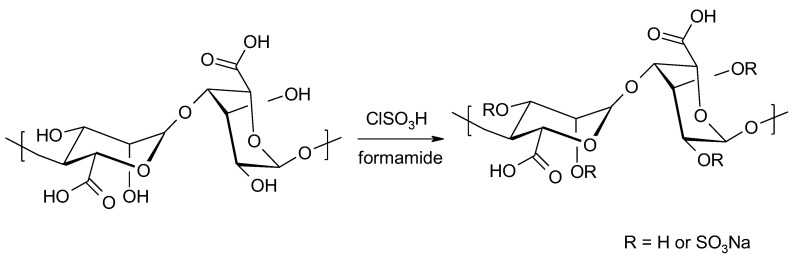
Preparation of sulphate alginate derivatives using chlorosulfonic acid.

The process of introducing sulphate residues into polysaccharides can be carried out by both chemical and enzymatic modification. Polysaccharide sulphates are biocompatible with blood and show anticoagulant activity [54,56]. The first method of incorporating sulphate groups into alginates was based on using chlorosulphonic acid and formamide as a solvent (Figure 8) [56]. The degree of substitution was 1.41.

**Figure 9:**
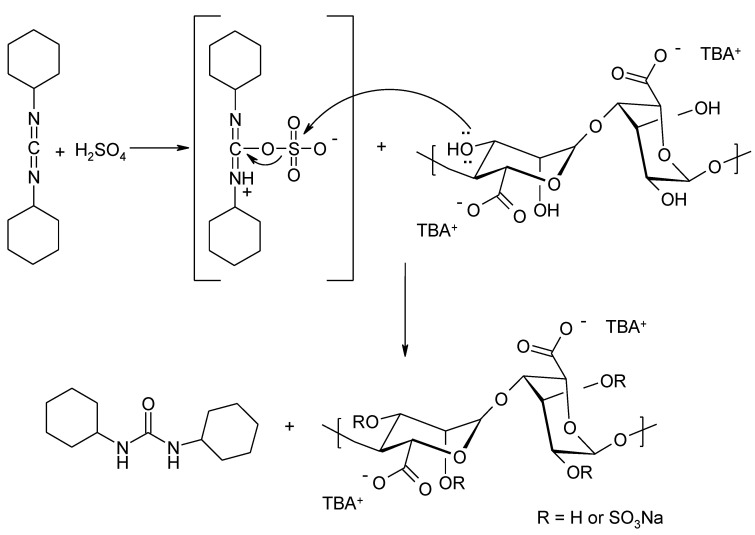
Formation of sulphate alginate derivatives using DCC and sulfuric acid.

Cohen et al. proposed a method of obtaining alginate sulphates using carbodiimide coupling reagents [57]. The hydroxyl groups were esterified with the DCC–sulfuric acid derivative generated in situ. No activation of alginate carboxyl groups was observed (Figure 9).

The DCC-H_2_SO_4_ adduct reacts with the nucleophilic hydroxyl groups of the alginate. The reaction products were characterized based on ^13^C-NMR. No change in the chemical shift was observed in the spectra for C-1 and C-6. It was found that sulphate esters are formed with either one or two hydroxyl groups at C-2 and C-3. The use of a strongly acidic reagent causes a significant reduction in the molecular weight of the polysaccharide. The average molecular weight decreases during the reaction from 100 to 10 kDa, but this has no effect on the M/G ratio.

**Figure 10:**
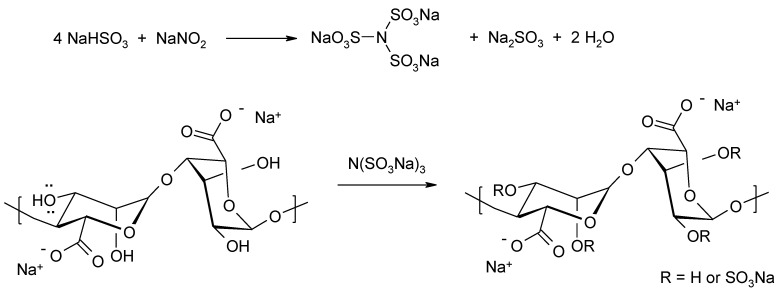
T Formation of sulphate derivatives of alginates using a mixture of NaHSO_3_ and NaNO_2_.

Traditional methods of forming alginate esters with sulfuric acid using sulfuric acid, chlorosulfonic acid, sulfamic acid, sulfuryl chloride, or sulfur trioxide cause degradation. A new synthetic method that can avoid degradation of the alginate was proposed by Fan et al. [58]. The solution uses sodium hydrogen sulphate IV and sodium nitrate III in water (Figure 10).

The DS value was 1.87 for a reaction performed at 40 °C, with a ratio of esterifying agent to uronic acid of 2:1. The anticoagulant properties of sulphated alginate derivatives were found to depend on the degree of DS, the molecular weight of the polysaccharide, and the concentration [59,60].

The most important use of sulfated alginate derivatives is due to the fact that they have properties similar to heparin [61,62]. Additionally, they were used to obtain biologically active conjugates with cell-adhesion molecules, growth factors, and chemokines [63,64,65]. Sulfonated alginates can be useful for treating viral infections caused by *Flaviviridae*, *Togaviridae*, *Rhabdoviridae*, and *Herpesviridae* [66,67,68]. It is believed that the negative charge resulting from the presence of carboxylic and sulphated residues is responsible for the antiviral activity, as it favors interaction with the positively charged host cell. As a result, contact with the virus and the host cell is difficult [69]. Another explanation for the observed antiviral activity relates to the inhibition of viral penetration, as alginates form a physical barrier around the cells [70]. Alginates with a high content of M blocks have immunomodulatory properties, resulting from the activation of macrophages responsible for the excretion of cytokines and cytotoxic factors [71].

### 3.2. Oxidation of Alginates and Use of Carbonyl Groups for Further Functionalization

**Figure 11:**
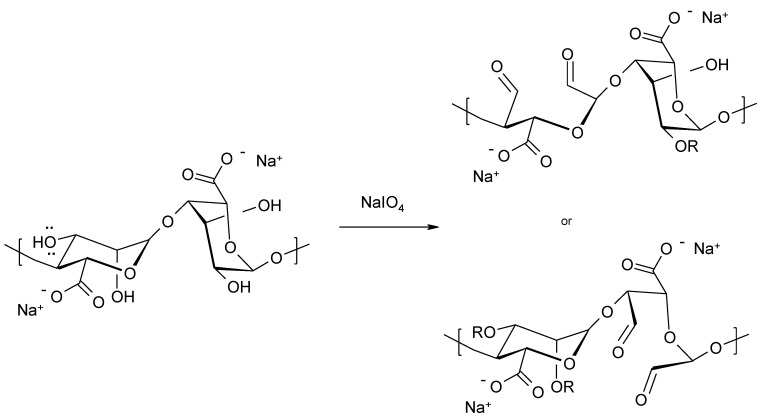
Oxidation of sodium alginate.

In recent years, there has been much attention given to the oxidation of alginate. Oxidized alginates contain more reactive functional groups compared to the original polysaccharide and are characterized by faster degradation [72]. This is beneficial when the alginates are used as carriers for controlled drug delivery [73,74]. By breaking the carbon-carbon bond, the oxidation of vicinal -OH groups at the C-2 and C-3 positions of sodium alginate with sodium periodate (Figure 11) leads to the formation of two aldehyde groups in each oxidized monomeric unit. This results in a more flexible polysaccharide chain and the incorporation of two reactive functional groups.

To reduce side reactions, it is crucial to protect the alginate against UV radiation and to control the degree of oxidation by varying the concentration of the oxidant. Controlling the degree of oxidation has proven to be particularly important, because alginates oxidized above 10 mol% do not gel in the presence of calcium ions [75].

**Figure 12:**
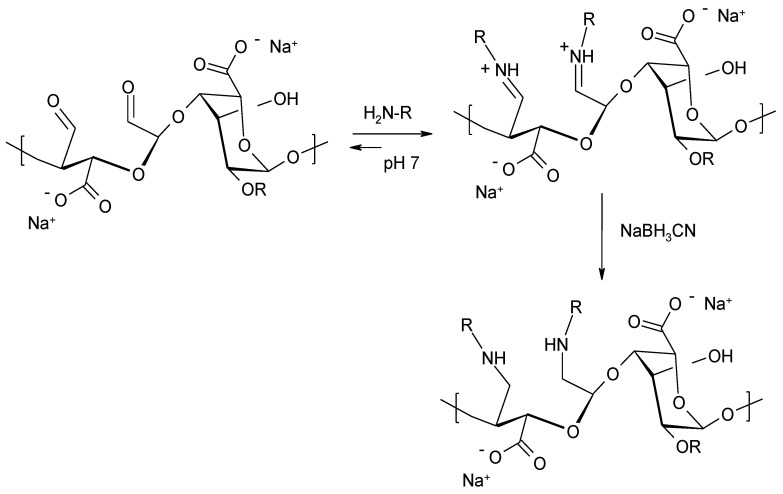
Reductive amination of oxidized sodium alginate.

The oxidized alginate with aldehyde groups in the polymer chain offers possibilities for further functionalization, in particular by reductive amination (Figure 12). Various aliphatic amines have been used for this purpose. It has been shown that NaBH_3_CN is a more effective reducing agent than NaBH_4_. The advantage of NaBH_3_CN is due to the fact that the reduction of imine groups using the anion of BH_3_CN- occurs quickly at pH 6–7, whereas the reduction of aldehyde or ketone groups in the same pH range is marginal [76,77,78,79,80].

The use of α-amino-ω-benzyloxytetraoxoethylene for reductive amination ((C_6_H_5_(OCH_2_CH_2_)_4_NH_2_, BzlO-TEG-NH_2_) results in an alginate derivative with much higher hydrophobicity than the original polysaccharide. At the same time, the carboxylic functions in the polysaccharide units are not affected, and the alginate maintains the ability to gel under calcium ions [72].

**Figure 13:**
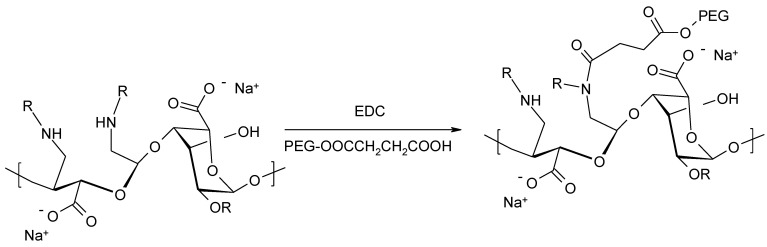
Synthesis of PEG-ylated alginate derivative.

Subsequent alginate derivatives were obtained by introducing a polyethylene glycol derivative into the secondary amine group formed as a result of reductive amination of the oxidized alginate (Figure 13) [81]. PEG-ylated alginate was obtained using a carbodiimide coupling reagent in an aqueous medium.

The obtained derivative retained the ability to gel in the presence of Ca^2+^ ions, due to the presence of free carboxyl groups of the polysaccharide.

Due to their faster degradation rate and higher content of reactive groups compared to native alginate, hydrogels based on oxidized alginate (OA) are used widely as biodegradable materials for tissue engineering applications. OA-based hydrogels are used in tissue engineering of bone, cartilage, blood vessels, cornea, and other soft tissues [82,83,84,85,86,87,88,89,90,91,92,93,94,95].

### 3.3. Chemical Modification of Alginated via Click Chemistry Reactions

Click chemistry reactions are another method for chemically modifying alginate derivatives. Click chemistry reactions take place under mild conditions, are not sensitive to presence of water, and do not require the use of complicated methods of isolating the final products. Click chemistry reactions lead to the formation of a stable conjugate. Additionally, click chemistry is characterized by high selectivity leading to formation for a single product [96]. Click chemistry reactions have also found application in the synthesis of modified biopolymers, including alginates. Due to specific requirements for each reaction in this group the appropriate alginate derivatives have to be applied (Table 4).

Although, materials obtained via click chemistry reactions were investigated to assess the possibility of the production of biomaterials from them, it was found that the obtained new alginate derivatives have application potential as materials for cell encapsulation, drug delivery, producing antimicrobial materials, tissue engineering, targeted delivery of systemic small molecules, and wound dressing [96].

### 3.4. Chemical Modification of Carboxyl Groups

**Figure 14:**
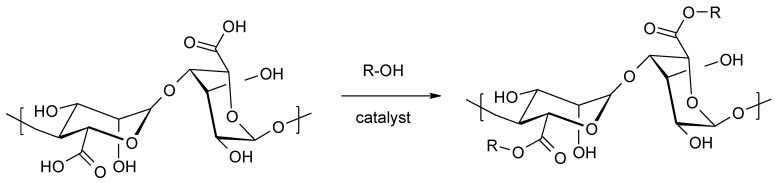
Esterification of alginates with alcohols.

The esterification reaction is often used as a simple method of attaching alkyl groups to a carboxyl function. This approach has been successfully applied to the modification of native alginate, thanks to which the hydrophobic character of the material increased [97,98,99]. Alginates can be modified by direct esterification by treatment with selected alcohols in the presence of a suitable catalyst (Figure 14).

For many years, the pool of commercially available alginate derivatives was comprised of only one ester derivative, which was formed by the reaction of alginates with propylene oxide as a result of epoxy ring opening [72]. The limited use of the esterification reaction as a method of alginate modification is related to the degradation of the polysaccharide chain in the presence of acids (catalysts of the classical esterification reaction) and to the quite drastic esterification reaction conditions necessary to obtain a high degree of conversion (acidic environment, increased temperature).

Broderick et al. [100] showed that it is possible to obtain alginate butyl esters by the reaction of sodium alginate, butanol, and H_2_SO_4_ as a catalyst at room temperature. The obtained alginate butyl esters were used for the encapsulation of hydrophilic and hydrophobic compounds. Esterification with butanol did not affect the loss of gelability in the modified alginate and did not cause material toxicity.

**Figure 15:**
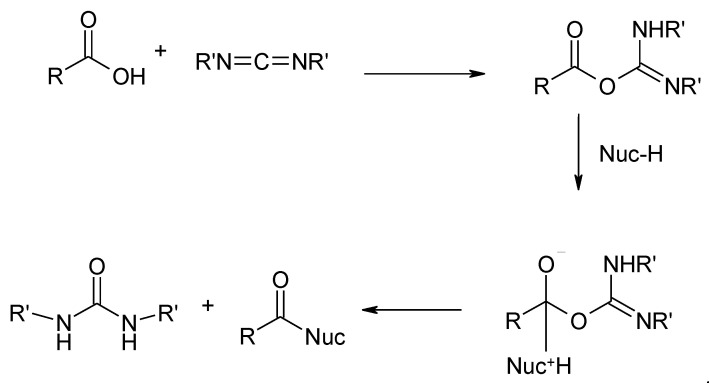
Activation of the carboxyl group using carbodiimide derivatives and subsequent reaction with nucleophilic reagents.

Extremely mild alginate esterification conditions can be achieved in reactions using coupling reagents. Carbodiimide derivatives are most commonly used for this purpose (Figure 15). Carbodiimide derivatives are used in the synthesis of alginate esters, as well as amide and peptide derivatives (Figure 15).

Water-soluble amphiphilic esters of cholesterol and alginates can be obtained by reacting sodium alginate, cholesterol, DCC as a condensing reagent, and DMAP as a catalyst at room temperature in a neutral environment [101]. The amphiphilic nature of the alginate cholesterol ester means that the obtained derivatives are self-assembled into stable nano-aggregates stabilized by intra- and intermolecular hydrophobic interactions between cholesterol residues.

**Figure 16:**
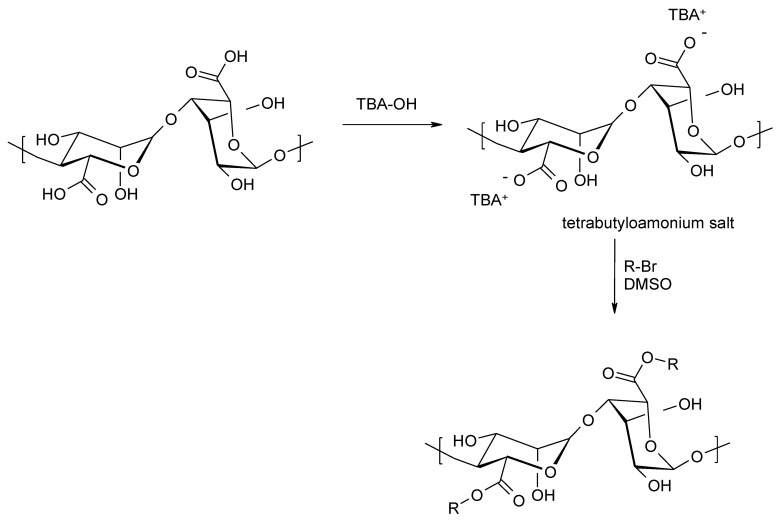
Synthesis of alginic acid esters using alkyl halides.

It is also possible to obtain alginate esters by reacting alkyl halides with tetrabutylammonium salts of alginic acid [102,103,104]. Esterification takes place under homogeneous conditions in organic solvents, since the transformation of alginic acid into tetrabutylammonium salts gives derivatives that are soluble in typical organic solvents (Figure 16).

The alkyl halide method has been used for the synthesis of structurally diverse esters of alginic acid. Its advantage is the possibility of also using long-chain alkyl halides. This enables hydrophobization of the surfaces of alginate materials [83,105]. The main drawback of the alkyl halide method is the possible alkylation of hydroxyl groups, which may ultimately lead to stable ether derivatives.

The chemoselective modification of the alginic acid esterification process proposed by Pawar and Edgar [105] involves the use of tetrabutylammonium salts of alginic acid and alkyl halides. Esterification was carried out in an organic solvent system (DMSO, DMF, DMAc or DMI) with the addition of tetrabutylammonium fluoride (TBAF) [20]. Partially and fully esterified benzyl, butyl, ethyl, and methyl esters of alginic acid were obtained by treatment with appropriate alkyl halides. Saponification reactions showed that the alkylation was completely chemoselective towards carboxyl groups (no alkylation products of hydroxyl groups).

**Figure 17:**
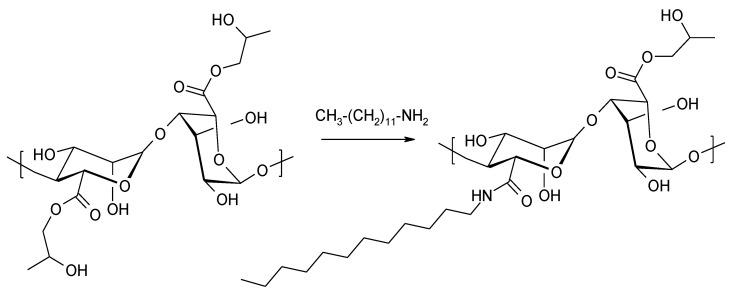
Synthesis of amides of alginates in the reaction of propylene glycol esters of alginates with amines.

Alginate propylene glycol esters can be used as substrates in the nucleophilic substitution reaction on the sp^2^ carbon of the ester group with alkylamines of various carbon chain lengths (C8, C12, C14) [106] (Figure 17).

The incorporation of hydrophobic domains into the surface of a hydrophilic alginate results in the formation of three-dimensional materials with amphiphilic properties [107,108,109,110].

**Figure 18:**
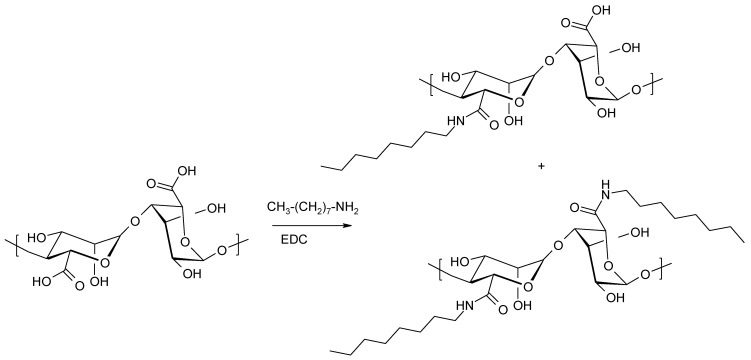
Formation of *N*-octylamide of alginic acid using EDC as a condensing agent.

Carboxylic acid functions are converted directly to amide groups. Typically, amidation is used to increase the hydrophobicity of the starting polysaccharide. Classical coupling reagents used in peptide synthesis can also be used in the formation of amide derivatives of alginic acid. To obtain alginate derivatives with *N*-octylamine, EDC-HCl (1-ethyl-3-(3-dimethylaminopropyl) carbodiimide hydrochloride was used [111] (Figure 18).

Water-soluble EDC-HCl has been used to obtain a variety of amide derivatives of alginic acid [112,113,114,115,116].

The attachment of highly hydrophobic amines (*N*-dodecylamine) to alginates leads to amphiphilic materials. The physicochemical properties of the materials result from the hydrophobic nature of the amide substituent and the hydrophilic nature of the polysaccharide matrix. Furthermore, 2-chloro-1-methylpyridinium iodide (CMPI) was used as the condensing reagent [117].

Alginate derivatives modified with amino acids, amines, sugars, and peptides have been obtained using both in situ formed (in reaction of CDMT and NMM) and commercially available 4-(4,6-dimethoxy-1,3,5-triazin-2-yl)-4-morpholine chloride (DMTMM) [118,119]. This triazine-based coupling reagent was found to be highly efficient. The alginate derivatives modified with amino acids and peptides showed much higher resistance to enzymes.

**Figure 19:**
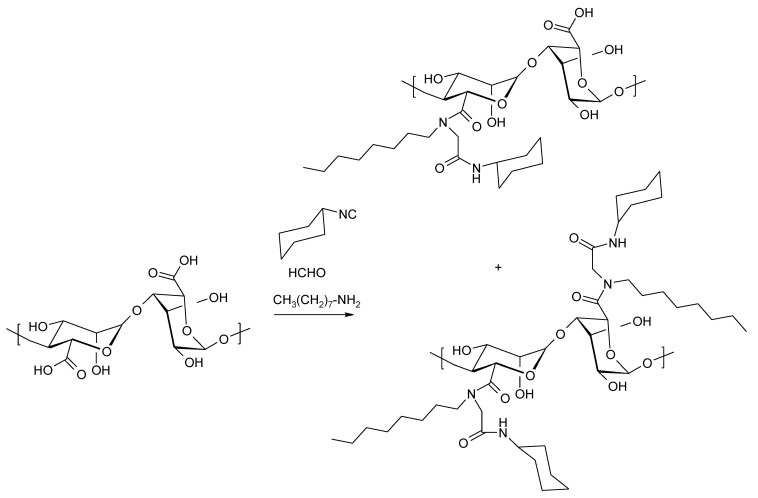
Ugi multi-component reaction for the preparation of amide alginate derivatives.

The multi-component Ugi reaction has also been used to obtain alginic acid amides, with carbonyl compounds (ketones or aldehydes), amines, isonitriles, and carboxylic acids used as the substrate (Figure 19).

This method is often used in combinatorial chemistry, making it possible to quickly harvest products, from which it is possible to select those with the desired biological activity. The use of alginic acid instead of classic carboxylic acids allows for the rapid synthesis of structurally diverse hydrophobic alginate derivatives [120,121,122]. The necessary condition for the success of the reaction is the conversion of sodium alginate to alginic acid (acidification with HCl to pH 3.6).

#### Reactions Using the Carboxyl Functions of Alginic Acid to Attach Biologically Active Ligands

Alginates are widely used biomaterials in bioengineering [123]. The most important requirement for biomaterials is their ability to provide a physically and chemically beneficial environment for living cells. To increase the interaction of the alginate matrix with cells, it is functionalized with cell-specific ligands or with extracellular signaling molecules. In addition to enhancing cellular interactions, functionalization may also play a role in controlling cell growth, differentiation, and behavior. Alginates have many advantages as biomaterials, including hydrophilicity, biocompatibility, and lack of immunogenicity [124]. The ability of alginates to form gels capable of encapsulating cells, drugs, and other biologically active compounds is another important factor. The most important disadvantage is that cells adhere relatively poorly to natural alginates [125]. For this reason, chemical functionalization with cell signaling compounds or adhesion enhancing compounds is crucial to overcome the low affinity of alginates to the cell surface. There are many examples of the functionalization of alginates with adhesion enhancing compounds.

The covalent attachment of amine galactose derivatives to obtain an increased affinity for hepatocytes, resulting from the specificity of the interactions between β-galactosyl derivatives and asialoglycoprotein receptor (ASGPR) on the surface of hepatocytes, is described in the literature [126]. Alginic acid was coupled with 1-amino-1-deoxy-β-d-galactose (Gal-1-NH2) using water-soluble carbodiimides as coupling reagents. Based on the 1H-NMR spectra, the DS obtained in a reaction with 1.5 equivalents of Gal-1-NH_2_ per unit of monosaccharide at pH 4.5 was 0.36. Another approach to the galactosylation of alginates was used by Akaike et al. [127]. Lactobionic lactone was first dehydrated and then coupled with ethylenediamine. The presence of a free amino group in the product allowed for coupling with alginic acid in the presence of EDC/NHS. The most commonly used method of alginate modification with peptides involves the activation of carboxylic acid with EDC (or another carbodiimide) and the subsequent formation of an active *N*-hydroxysuccinimide ester, which is the actual acylating reagent for free amino groups in a peptide.

Alginate modifications with bioactive peptides have traditionally been performed using carbodiimides. It has been shown that the average coupling efficiency varies between 0.1 and 1.0 mol% of the peptide per uronate monomer [128]. Although this concentration of peptides induces deposition and the interaction of modified alginates with myoblasts [129], olfactory cells [130], mesenchymal stem cells [131], and endothelial cells [132]; increasing the concentration of peptides increases both cell binding and differentiation, as demonstrated by Rowley and Mooney using C2C12 myoblasts and RGD peptide modified alginate. A similar result was observed in the case of YIGSR peptide-modified alginate and neuroblastoma cells [133]. Increasing the degree of peptide substitution is important in the preparation of peptide-modified alginates for use as bioactive hydrogels and coating materials [134]. The use of carbodiimides as coupling reagents is also associated with the formation of urea and *N*-acylurea derivatives, which impair the gelling properties and biological activity of the peptide.

Most significantly, with a low degree of substitution, the carbodiimide coupling reaction is more selective for G residues than M residues, and the G residues adjacent to M residues are more reactive than G residues adjacent to G residues. Reducing the amount of free G residues lowers the ability of the modified alginates to cross-link and form gels by divalent ions. The proposed solution is the use of mannuronate chemoenzymatic modification [135]. In the first step, the mannuronate is conjugated with a peptide ligand (GRGDYP) using carbodiimides. The second step is the synthesis of alginates catalyzed by recombinant epimerases AlgE4 and AlgE6 from *Azotobacter vinelandii* or another bacterium [136]. This allows the introduction of G residues (gelling residues) into the polymer. Only the residues to which the bioactive peptide has not been attached undergo enzymatic reactions. Another proposed approach involves peptide coupling by using carbodiimides to alginate partially oxidized by periodate, followed by reductive amination. In this approach, the carboxyl group of the biologically active peptide is activated by the carbodiimide coupling reagent and coupled with the free amine group of the modified alginate [25]. Optimization using tyrosine methyl ester resulted in a repeatable synthetic protocol, which uses pic-BH3 (2-picoline-borane complex) as the reducing agent. The coupling efficiency was also dependent on the alginate composition, but to a lesser degree. The highest coupling efficiency was obtained using mannuronate. Three different bioactive peptides, GRGDYP, GRGDSP, and KHIFSDDSSE, were coupled to partially (8%) oxidized alginate. The degree of peptide substitution was in the range of 3.9–6.9%. Studies of cell adhesion by mouse myoblast cells (C2C12) and human tooth stem cells (RP89) to gels containing different amounts of alginate coupled with GRGDSP showed improved adhesion compared to the unmodified alginate. However, for RP89 cells it was necessary to use alginates with a higher peptide content.

**Figure 20:**
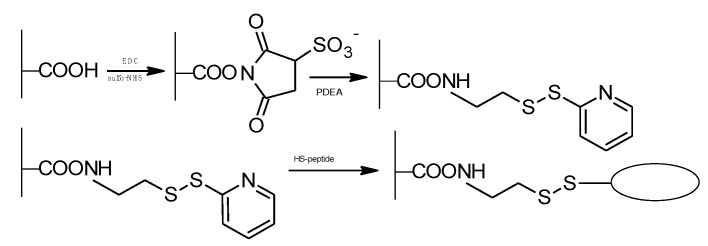
Synthesis of alginate-peptide conjugate by alginate-S-S-y intermediate.

Even though the water-soluble carbodiimide coupling method is well known and effective, useful modifications or alternative methods are still being sought, without the disadvantages of the EDC/NHS method. Bubenikova et al. [137] described a method to crosslink alginates with thiol-terminated peptides using the modified EDC coupling method with *N*-hydroxysulfo-succinimide (sulfo-NHS). In the next step, the heterobifunctional linker (2-(2-pyridyldithio)ethyleneamine) was introduced into the solution to form alginate-S-S-pyridine (Py) intermediate. After the addition of thiol-terminated peptide, thiol disulphide exchanges the pyridyl group to form an alginate-S-S-peptide conjugation (Figure 20).

Biological assessment of the products showed a lack of alginate-S-S-py-related toxicity [137].

In 2012, Guo et al. synthesized conjugates of alginate with H_2_N-Gly-Tyr-Ile-Gly-Ser-Arg-Gly-COOH, (GYIGSRG/GG) through tetrabutyloamonium alginate salt formed during neutralization of alginic acid with tetrabutyloamonium hydroxide. Carboxylic groups were then activated with 2-chloro-*N*-methylpyridine and peptide was added with TEA. The reaction resulted in directly bonded alginate-peptide conjugate [138]. Another way to connect peptides with alginates is to use adipic acid dihydrazide as a linker. In the first step, non-activated alginate or alginate activated with cyanogen bromide was reacted with adipic acid dihydrazide (ADH). The intermediate product was reacted with the peptide in carbodiimide mediated coupling, resulting in alginate-ADH-peptide conjugate [139]. It is also possible to modify alginate itself, to enable bonding of other biomolecules. By oxidizing sodium alginate to alginate di-aldehyde (ADA) with periodate [140], it is possible to cross-link ADA hydrogel with peptides or proteins, without any additional coupling reagents [141].

There have been many efforts to obtain new conjugates composed of alginates and biologically active compounds, especially of natural origin. Zhu et al. synthetized a pericellular matrix-like material using alginate-amino acid derivatives in combination with poly(dl-glycolic acid). To immobilize four amino acids (l-lysine, l-arginine, l-aspartic acid, L-phenylalanine) on the surface of the alginate, a water-soluble derivative of carbodiimide was used. The percentage of amino acids on the alginate surface was in the range of 2.14–3.0. The alginate in the membrane form has been tested to assess the attachment and growth of chondrocytes. In comparison with a virgin PDL-LA membrane, membranes containing alginate-amino acid show the same or improved ability to attach chondrocytes and, except in the case of the membrane with phenylalanine, the modified alginate accelerated cell growth. The best results were obtained for alkaline amino acids, such as arginine and lysine [115]. Numerous studies have investigated the bioactivity of alginate-peptide/protein conjugates and shown their usefulness in a range of potential applications. Examples are presented in Table 5.

Alginate materials differ in structure and physical properties. Size of the materials range from nano scale <0.2 µm to micro from 0.2 to 1000 µm, to macro scale >1 mm. Mechanical properties are dependent on the crosslinking agent and chemical modification of the alginate structure, but polymer itself is characterized with good strength, elasticity, high viscosity, and stability. Alginate materials occur in three main structure types: hydrogel adjusting to the shape of the mold, fibers, and beads [170,171].

## 4. Application of Alginate-Based Materials

Alginate derivatives are an attractive biomaterial for numerous applications, including tissue engineering, cell encapsulation, and wound healing [172,173,174,175], in situ formation of gels, controlled drug delivery and release systems, as well as targeted drug delivery [176,177,178,179]. The pool of pharmaceutical products based on alginates includes:

- Drugs administered orally (Gastrotuss baby syrup [180], Algicid suspension/tablets [181], Gaviscon Double Action Liquid [182] and Tablets [183]), creating a mechanical barrier between the stomach and esophagus that prevents reflux, choking, dysphagia, heartburn, belching, and irritability, which accelerates the movement of the stomach and regenerates the mucous membranes of the esophagus
- Materials applied to the skin (Flaminal Forte gel [184], Purilon Gel gel [185], Saf-Gel gel [186], Hyalogran dressing [187], SeaSorb dressing [188], Tromboguard dressing [189,190], Fibracol Plus dressing [191], Algivon dressing [192], Guardix-SG [193,194]), which affect dissolution of the dry layer and necrotic tissue, ensure a moist environment at the wound surface, have hemostatic and antibacterial activity, and influence tissue granulation, epithelialization, and healing
- Rectal agents (Natalsid suppositories [195]) used for chronic hemorrhoids, proctitis, and chronic anal fissures after rectal surgery
- Periodontal agents (Progenix putty, Progenix plus injection [196]), used for bone defects, and Emdogain gel [197,198] used for intraosseous defects and defects of mandibular furcation with minimal atrophy of the interdental bone
- Agents applied arthroscopically (ChondroArt 3D injection [199]), used in degenerative diseases of the joints and spine.

Numerous studies have shown that alginate derivatives have antibacterial, antiviral, and antifungal properties [200]. However, the mechanism of antimicrobial action is still unknown. It is believed that their antimicrobial activity may be due to the fact that negatively charged alginates interact with bacterial cells, leading to disruption of the cell wall/membrane and leakage of intracellular substances [201]. Membranes, caused by the formation of a sticky layer of alginate around the bacteria, make the transport of nutrients to the bacterial cells difficult [202]. Alginates have been found to have bacteriostatic activity against *Pseudomonas*, *Escherichia*, *Proteus*, and *Acinetobacter* [203,204]. The antimicrobial activity of alginates depends on their molecular weight, M/G block ratio, structural modifications, the pH of the environment, and the type of formulation use [205].

Calcium alginate activates platelets and affects thrombin, making it an effective hemostatic [206,207] and suitable for use in wound dressings [208,209,210]. Low-molecular-weight sodium alginate lowers blood pressure [211]. The hypotensive mechanism is due to the antagonism of voltage-gated calcium channels [212]. Potassium alginate is considered a promising agent for preventing cardiovascular complications related to hypertension, including hypertrophy of the heart and kidneys, and the risk of stroke [213].

Low molecular weight alginates have anti-oxidative and anti-inflammatory effects, which are associated with a reduction in the biosynthesis of nitric oxide, reactive oxygen species (ROS), prostaglandin E2, and COX-2 cyclooxygenase [214,215]. Their anti-oxidative activity may be related to the stimulation of monocyte secretion by anti-inflammatory cytokines, which is associated with alginates that have a high content of M residues [216]. Due to their ability to chelate, alginates can bind toxins and heavy metals, which helps protect against carcinogenesis [217,218].

Alginic acid has also been shown to be anti-anaphylactic, inhibiting the release of histamine from mast cells and reducing the expression of histidine decarboxylase and pro-inflammatory cytokines [219,220].

Alginates are considered promising candidates for use in preparations for the treatment of obesity and type 2 diabetes. They attenuate the postprandial glycemic response, by modulating gastric emptying [221] or inhibiting glucose transporters, and also affect the rate of intestinal glucose absorption [222]. The hypoglycemic effect of alginates may be related to a reduction in the activity of α-amylase, an intestinal enzyme responsible for the hydrolysis of bonds between glucose residues in carbohydrates [223,224,225].

Thanks to their fiber-forming properties and ability to form gels, alginates can mimic the structure of the extracellular matrix, and therefore provide a relatively neutral and moist microenvironment. The usefulness of alginates as macroporous scaffolds conditioning favorable conditions for cellular attachment, proliferation, and differentiation is evidenced by the presence on the market of two 3D products: AlgiMatrix (Thermo Fisher Scientific/Life Technologies, Carlsbad, CA, USA) and NovaMatrix 3D (NovaMatrix, Sandvika, Norway) [226,227]. To improve alginate biocompatibility, studies have been conducted on chemically modified alginate and its impact on cell function [21,44,228,229,230,231].

Scaffolds for bone tissue engineering must have a highly porous structure, to ensure a biological environment that promotes cell attachment and proliferation and tissue growth, as well as enabling nutrient flow. The levels of new bone formation and vascularization are considered critical factors for clinical applications of bone tissue engineering scaffold implants. Yingying Wang et al. studied an RGD-grafted oxidized sodium alginate/*N*-succinyl chitosan (RGD–OSA/NSC) hydrogel as a scaffold, with low-intensity pulsed ultrasound (LIPUS) applied as mechanical stimulation to achieve a high level of new bone formation and vascularization [232]. Muscle satellite cells induced by platelet-rich plasma encapsulated into 3-dimensional alginate hydrogel were found to enhance the production of tissue engineered bone, both in vitro and in vivo [233]. New alginate microparticle scaffolds and microfiber aggregated scaffolds were synthetized and characterized through in vitro studies [234]. The scaffolds demonstrated good mechanical and morphological properties. Their porous structure allowed vascularization and oxygenation, as well as cell migration, adhesion, and proliferation, making them suitable for use as bone substitutes. An alginate-Matrigel matrix containing isolated ovarian cells was developed to provide an environment in which isolated follicles and ovarian cells can survive and grow, creating a biodegradable artificial ovary. After transplantation, the alginate-based matrices were able to degrade, allowed vascularization, and elicited a low inflammatory response. They also supported cell survival and proliferation [235].

A very common modification of alginate in the tissue engineering field is the introduction of bioactive ligands [110,124,125,236,237]. The addition of such peptides, usually in the form of carboxyl-linked side chains, provides binding sites for cells that alginates lack. Yuji Yamada et al. investigated the use of three laminin active peptide-conjugated alginate matrices and their biological functions as biomaterial for tissue engineering. Depending on the amount of alginate, the matrices effectively promoted cell attachment, cell spreading with well-organized actin stress fibers, and neurite outgrowth [129]. In another study, chemospecifically functionalized alginates with thiol-ended peptides were assessed by in vitro assays. The alginate-based matrices with multiple peptide signals were found to promote specific cell interactions. It is possible that they could be further combined to generate coatings or complex hydrogel compositions, which could be used in tissue regeneration. A preliminary assessment of calcium hydrogels based on alginate–c(RGD) demonstrated the potential of the bioconjugated alginate–peptides as materials able to specifically interact with osteoblastic cell [115]. Alginate incorporated with the fluorenylmethoxycarbonyldiphenylalanine (FmocFF) peptide led to the production of a rigid, yet injectable composite hydrogel, without the addition of cross-linking agents, which could serve as a potential biomaterial for bone regeneration. Scanning electron microscopy revealed a nanofibrous structure, which mimicked the natural bone extracellular matrix. In vitro biocompatibility tests carried out with MC3T3-E1 preosteoblast cells demonstrated good cell viability and adhesion to the hydrogel fibers [130]. Alginates modified with peptides containing RGD (arginine–glycine–aspartic acid) or PHSRN (proline–histidine–serine–arginine–asparagine) sequences from fibronectin were mixed at different ratios to study possible additive and synergistic effects on adherent cells. Gels composed of RGD- and PHSRN-modified alginates showed enhanced cell differentiation and proliferation. Depending on the placement of the cell culture in the gels and the peptide ratio in the gels, calcium deposition showed different tendencies. Modifying these biomaterials could therefore be a way to develop scaffolds for bone tissue engineering, providing three-dimensional cell culture systems that more closely mimic the environment of the human body [238].

Modification of alginates is particularly attractive in the field of wound healing. A common approach is to produce alginate or alginate-chitosan fibers with bioactive properties, such as inherent antimicrobial activity [132,239,240]. A novel hybrid peptide, SIRVXVXPG (X: A or G), was designed from the laminin-derived peptide, SIKVAV, and an elastin-derived peptide, VGVAPG. The peptide was used to develop a new alginate dressing. Examination with a rabbit ear skin defect model in vivo showed very good epithelialization and contributed to producing a large volume of regenerated tissue. The new alginate dressings linked with the hybrid peptides are especially promising for the treatment of wounds with impaired healing [136]. For full-thickness wound healing, a hybrid hydrogel dressing composed of collagen, sodium alginate, polymyxin B sulfate, and bacitracin was developed. The hydrogel was effective against *E. coli* and *S. aureus*, and promoted cell growth and angiogenesis in a rat model by accelerating reepithelialization, collagen deposition, and angiogenesis [241].

Current restrictions on the use of antibiotics associated with increases in bacterial resistance require new solutions, including materials with antibacterial properties. Copper alginate conjugates with RGD derivatives obtained by deep spraying and covalent attachment of the peptide to the polysaccharide matrix are characterized by high antibacterial activity [242]. Cu-Alginate-RGD (conjugate with a chemically bonded peptide) has been found to have antibacterial activity against *Staphylococcus aureus* ATCC 6538 and *Klebsiella pneumonie* ATCC4352, at a level of A = 2.90 for *S. aureus*, which indicates significant antibacterial activity, and A = 4.56 for *K. pneumoniae*, which indicates strong antibacterial activity. Cytotoxicity studies on the L929 mouse fibroblast cell line using the Presto Blue test showed that copper alginate conjugates with RGD derivatives are not cytotoxic. Given that they also meet the requirements of biocompatibility and antibacterial activity (there is no need to introduce additional compounds to provide antimicrobial activity), materials based on copper alginate conjugates with biologically active peptides have great potential for use in regenerative medicine.

Alginates have also been used to encapsulate genetically modified fibroblasts. This could be an effective strategy for delivering therapeutic products to spinal cord injuries, while providing a suitable environment for host axon growth in the absence of immune suppression [243,244]. To improve this strategy, the alginate gel surface was modified either by coating it with laminin or by covalent attachment of a YIGSR peptide to the carboxylic acid groups on the alginate. The results showed enhanced cell adhesion and regeneration of the axons in the injured spinal cord. Peptide-modified alginate gels allow adhesion of NB2a neuroblastoma cells and promote neurite outgrowth when attached to the peptide-modified alginate surface [128]. Nanoparticles (NPs), in particular magnetic particle imaging (MPI) [245] coated biomacromolecules, including alginate [246], are considered a promising non-invasive imaging technique. Proper selection of the MPI coating biopolymer can give unique properties, including high selectivity for specific places in the body and low cytotoxicity.

## 5. Summary

Alginates are valuable biomaterials for many biomedical and pharmaceutical applications, in particular wound dressings, drug delivery, in vitro cell cultures, and tissue engineering. This is mainly due to their biocompatibility, mild gelling conditions, and simple modification methods, leading to the production of alginate derivatives with new properties. Despite the development of physical, chemical, and enzymatic methods of alginate modification, there is still a need for new solutions to obtain materials with properties tailored to specific applications. It seems that in the near future there will be much wider use of alginate-based materials, because it is already possible to obtain derivatives containing more than one active substance. This will allow for simultaneous action on various therapeutic targets, more precise delivery of active substances, extended duration of action, and prolonged or sequential release in response to external environmental changes (chemical, mechanical, magnetic, and other signals). The introduction of factors into the alginate matrix to determine its interaction with cells is a key requirement for many tissue engineering applications. The type of adhesion ligands and their spatial organization in the alginate matrices are important variables, as they can regulate the phenotype of the cell and thus the final function of regenerated tissues. Until now, RGD peptides have been used most widely as cell adhesion ligands. However, there is now the possibility of either using many ligands, combinations of ligands, or both, which could improve the production efficiency of replacement tissues and organs. Further research is necessary to better understand the properties of alginates and their derivatives, which would allow the development of new alginate gels with improved interaction with various types of cells and tissues. In the near future, the ability to create new alginate derivatives by means of precise physical, chemical, and enzymatic modifications could allow use of alginates in personalized therapies and diagnostics. The production of new alginate derivatives with different properties, using genetic engineering to control the biogenetic synthesis of the alginate by bacteria, remains a significant challenge.

## Figures and Tables

**Table 1:** Solubility of alginates in various solvents (c = 15 mg/mL).

	H_2_O	EG	DMAc	DMF	DMSO	DMAc /LiCl	DMF /TBAF	DMSO /TBAF	DMAc /TBAF	DMI /TBAF
H-Alg	−	−	−	−	−	−	−	−	−	−-
Alg-Na	+	−	−	−	−	−	−	−	−	−
Alg-TBAF	+	+	−	−	−	-	+	+	+	+

H-Alg—alginic acid; Alg-Na—sodium alginate; Alg-TBA—tetrabutylammonium alginate; EG—ethylene glycol; DMAc—*N*,*N*-dimethylacetamide; (+)—complete solubility; (−)—partially soluble or insoluble.

**Table 2:** Substitution ratio of acetyl residues in blocks M and G.

Origin of Alginate	DS	Acetylation (%)	
		M	G
*L. hyperborea*	0.1	>90	<10
*L. hyperborea*	0.4	80	20
*A. nodosum*	0.4	3	36

**Table 3:** DS value as a function of the alginate structure and acetylation reaction conditions.

Substrate	DS (Homogeneous Reaction)	DS (Heterogeneous Reaction)
100% M	0.80	0.91
63% M	0.80	0.48
29% M	0.78	0.63
13% M	0.78	0.54
0% M	0.82	0.72

DS derived from 1H-NMR. Alginate TBA salts were used as the substrate at a concentration of 100 mg/mL. Under heterogeneous reaction conditions, the solvent was DMSO. Under homogeneous reaction conditions, the solvent was 100 mg TBAF/mL DMSO.

**Table 4:** Examples of using the click chemistry reaction to obtain new alginate derivatives.

Click Reaction	Functional Groups Involved in Reaction	Characteristic
Copper-(I)-Catalyzed Azide-Alkyne Cycloaddition (CuAAC)	Azide–Alkyne 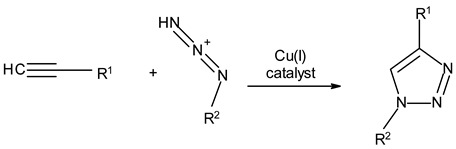	Cu-catalyzed (cytotoxic and difficult to remove from product) Reversible Bioorthogonal No side product
Strain-Promoted Alkyne-Azide Cycloaddition (SPAAC)	Azide–cyclic Alkyne 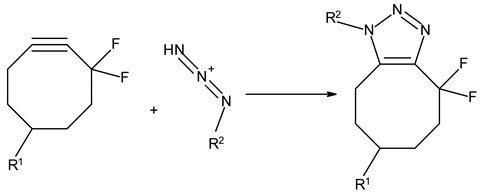	No catalyst needed Hydrophobicity of alkyne ring hindering the reaction
Inverse Electron Demand Diels–Alder Cycloaddition (IEDDA)	Dienophile–Diene 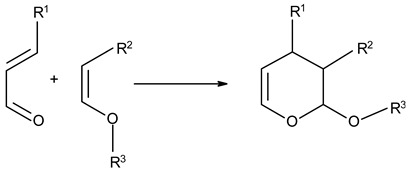	No catalyst needed
Diels–Alder Reaction	Diene–Alkene 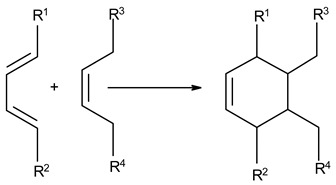	No catalyst needed Thermally reversible
Thiol-Ene Addition	Alkene–Thiol 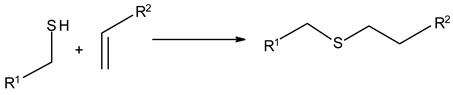	Photoinitiator needed Spatial and temporal control
Thiol-Michael Addition Click Reactions	Thiol–α,β-Unsaturated carbonyl compound 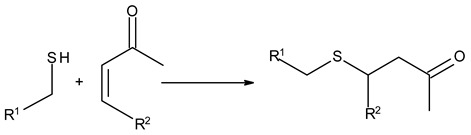	No catalyst needed Mild conditions
Thiol-Yne Addition	Alkyne–Thiol 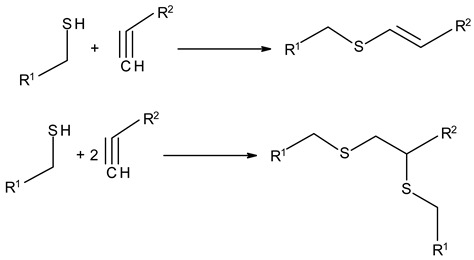	Initiator needed
Oxime Coupling	Aminooxy compound–Aldehyde/Ketone 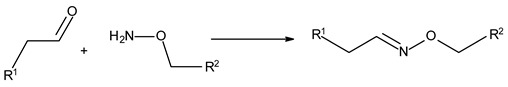	No catalyst needed Fast reaction under mild conditions

**Table 5:** Summary of studies on the biological activity of alginate-peptide/protein conjugates.

Peptide/Protein	Origin of Peptides	Bonding to Alginate	Material Morphology	Biological Impact		Ref.
RGD	Fibronectin	Directly by EDC coupling	Printed hydrogel	Neuronal cells, increased neurite growth		[142]
		Directly by EDC coupling	Hydrogel layer	Increased adhesion to the matrix		[110]
			3D beads	Increased viability in a short time, lack of enhanced survival		
		Directly by EDC coupling to methacrylate alginate	Hydrogel	Modified alginate promoted chondrocyte adhesion and spreading on the surface of the hydrogels		[143]
		Directly by EDC coupling on macroporous alginate scaffold	Hydrogel	Increased proliferation of MSCs in chondrogenic medium		[144]
GGGGRGDY (RGD)		Directly by EDC coupling	Hydrogel	Increased cell attachment to the matrix, increased cell survival and recovery, inducing the organization of cardiac muscle tissue		[145]
G6KRGDY (RGD)		Directly bonding self-assembling peptides by EDC coupling	Hydrogel	-		[146]
A6KRGDY (RGD)						
V6KRGDY (RGD)						
GGGGRGEY	HCV E2 glycoprotein	Directly by EDC coupling	Solution	Less effective at attenuating scar expansion compared to unmodified alginate, similar myofibroblast infiltration into the scar compared to different biomaterial-treated infarcts	Lack of cardiofibroblast attachment to matrix	[147]
GGGGRGDY/YIGSRYIGSRY (RGD/YIGSR)	Fibronectin/Laminin				Strong interaction of cardiofibroblast with the matrix	
YIGSR	Laminin	Directly by EDC coupling	Gel	Allowed adhesion of cells to the peptide-gel conjugate and promoted neurite outgrowth from the attached NB2a cells		[148]
YIGSR	Laminin	Directly by EDC coupling	Printed hydrogel	Neuronal cells, increased neurite growth		[122]
RGD-YIGSR	Fibronectin, Laminin					
GYIGSRG	Laminin	Directly via tetrabutyloamonium alginate salt, followed by 2-chloro-*N*-methyl pyridine/TEA coupling	Hydrogel	Induced angiogenesis 14 days after implantation and increased neovascular density almost 1.5 times in comparison to the control		[118]
AGTFALRGDNPQG	Laminin	Directly by EDC coupling	Matrice0073	Promoted integrin avb3-mediated cell attachment and neurite outgrowth		[149]
RKRLQVQLSIRT				Promoted syndecan-mediated cell attachment, lower amounts of peptide-alginate showed strong neurite outgrowth activity decreasing with increasing amounts of peptide-alginate		
ATLQLQEGRLHFXFDLGKGR, X: Nle				Promoted strong cell attachment, promoted extensive cell spreading of human dermal fibroblasts		
ERRANAVRDVLVNEY	Outer membrane proteins of *Pseudomonas aeruginosa*, Position 294–308	Adipic acid dihydrazide as linker	-	Immunogenicity, Higher levels of IgG antibodies in comparison to alginate and native peptide 294–308		[119]
AGLGVGFNFGGSKAA	Outer membrane proteins of *P. aeruginosa*, Position 176–190			Immunogenicity, no difference between conjugate and alginate or native peptide 176–190		
Fmoc-FF-OH	Self-assembling peptide	Alginate-Fmoc-FF composite	Hydrogel	High biocompatibility confirmed with osteoblasts, properties similar to a natural extracellular matrix		[150]
LL-37 peptide	Native	Mixture	-	Reduced toxicity to mammalian cells while maintaining antibacterial properties		[151]
Tet213	Modified	Directly by EDC coupling	ALG/HA/ COL-Tet213 3D-porous dressing	Improved antimicrobial activity, tested against MRSA, *Staphylococcus aureus*, *Escherichia coli*, confirmed biocompatibility		[152]
EWGRRMMGWGRGRRMMRRWW (Ib-M6)	Ib-AMP4 analogue	Peptide encapsulated in PVA-Alg pellets	Polyvinyl alcohol- alginate (PVA-Alg) matrix	Inhibited growth of microorganisms		[153]
GQGFSYPYKAVFSTQ sequence)	Bone forming peptide-1	Peptide incorporated in the structure	Porous scaffold with incorporated peptide	Positive impact on cell adhesion, proliferation and aggregation towards MG-63 cells in vitro		[154]
GGGGGHKSP (GHK)	Native peptide analogue	Bonding to aldehyde group at oxidized alginate	Hydrogel	Increased proliferation and viability of the cells, ability to stimulate osteogenic differentiation of MSCs		[121]
Gelatin- GGGGGHKSP (gelatin-GHK)	Native protein-native peptide analogue					
REDV	Fibronectin	-	Hydrogel	Increased proliferation and enhanced angiogenesis		[155]
Ac-KSIRVAVAPG *	Hybrid peptide laminin/Elastin	Cross-linked by ethylenediamine fibrous calcium alginate with peptide directly bonded by EDC coupling	Fibrous dressing	Induced cell attachment, effective promotion of granulation tissue regeneration and epithelialization		[156]
Ac-KSIRIAIAPG *				Lower activity than other peptides studied in this research		
Ac-KSIRIAIAPG *				Induced cell attachment, effective promotion of granulation tissue regeneration and epithelialization		
Ac-KSIRIGIGPG *				Slightly increased proliferation in comparison to control		
Ac-KSIKVAV (SIKVAV) *	Laminin			No significantly enhanced activity		
Ac-KVGVAPG (VGVAPG) *	Elastin					
Human serum albumin	Native	Hydrogel microspheres encapsulating a FITC-KRFK peptide coated with a human serum albumin-alginate membrane (HSA linked to ester groups of PGA by amide bonds)	Microspheres made of Ca^2+^ cross-linked PGA and alginate (2:1)	Surface membrane survived citrate treatment and lyophilization, no cytotoxicity against osteoblasts, slow release of the bioactive peptide from the core		[157]
		Hydrogel microspheres encapsulating KRFK peptide coated with a human serum albumin-alginate membrane (HSA linked to ester groups of PGA by amide bonds)		Gel strength did not influence the amount of the peptide, the size of the microspheres did not influence affinity for the peptide biding sites, unlabeled KRFK peptide appeared to release significantly faster from microspheres, properties such as size, charge, and hydrophilicity impacted the time needed to release the peptide from the microspheres		[158]
c(RGD)	Fibronectin	Peptide bound to alginate via amide bond of heterobifunctional linkage, peptide connected via disulphide bond	Hydrogel	No cytotoxicity and increased ability to attach osteoblasts		[117]
FHRRIKA	Bone sialoprotein			No cytotoxicity		
KRSR	Fibronectin, vitronectin, bone sialoprotein, thrombospondin, osteopontin					
CGGREDV	Fibronectin	Multivalent surface – thiol modified alginate bonded with gold nanoparticles by Au-S bond, peptide bonded to gold particles on the surface of AuNPs	Hydrogel	Multivalent ligand maintained EC selectivity of the REDV peptide, compared with the monovalent ligand the REDV cluster showed superior EC adhesion capability, the multivalent ligand more efficiently promoted angiogenesis in vivo, the density of a new blood vessel in hydrogel containing the multivalent ligand was approximately 20% higher than that of the monovalent scaffold		[159]
GREDV		Monovalent surface–peptide connected with alginate by amide bond				
Collagen originated peptides	Collagen	Directly by EDC coupling	-	Showed good hydrogen peroxide scavenging activity and promoted cell growth		[160]
Oligoproline derivatives	PPI/PPII helical structure	Directly by DMT/NMM/TosO-	Calcium alginate nonwoven modified with oligoproline derivatives	No cytotoxicity		[161]
Gelatin	Native	Hydrogel loaded with microbeads of blended gelatin-pectin	Hydrogel loaded with microbeads	Enhanced stem cell function and osteogenic differentiation capability		[162]
Gelatin methacrylate RGD	Gelatin	Alginate-RGD hydrogel with encapsulated gingival mesenchymal stem cells	Hydrogel	Accelerated wound closure and healing without infection		[163]
Interleukin-4	Native	Hydrogel complexed with alginate-chitosan microspheres loaded with interleukin-4 and endothelial progenitor cells (EPCs) and RAW264.7	Hydrogel loaded with microbeads	Promotes microvascularization		[164]
Gelatin methacryloyl	Gelatin	Alginate/Gelatin methacryloyl hydrogel loaded with hydroxyapatite	Hydrogel	Enable cell proliferation, spreading and adhesion. Promote the osteogenic differentiation of MC3T3- E1 cells		[165]
Vascular endothelial growth factor (VEGF), Platelet-derived growth factor (PDGF)	Native	VEGF and D+PDGF nanoparticles encapsulated in calcium alginate hydrogel	Hydrogel loaded with	Improvement of vascularization and vascular maturity in testicular tissue grafts		[166]
Fibrin	Native	Fibrin/alginate scaffold with incorporated calcium phosphate deposits	Foam	Biocompatible and proangiogenic		[167]
Collagen	Native	Alginate/collagen hydrogel bead containing lapidated tissue factor	Hydrogel beads	Hemostatic activity, undetectable cytotoxicity		[168]
Laminin Gelatin	Native	Mixed oxidized alginate-gelatin-laminin hydrogel	Hydrogel	Increase in neuronal differentiation in comparison to oxidized alginate-gelatin, with enhanced neuron migration from the neurospheres to the bulk 3D hydrogel matrix		[169]

* Each of these peptides independently promoted proliferation.
